# Gelatin-Methacryloyl (GelMA) Hydrogels with Defined Degree of Functionalization as a Versatile Toolkit for 3D Cell Culture and Extrusion Bioprinting

**DOI:** 10.3390/bioengineering5030055

**Published:** 2018-07-18

**Authors:** Iliyana Pepelanova, Katharina Kruppa, Thomas Scheper, Antonina Lavrentieva

**Affiliations:** Institute of Technical Chemistry, Gottfried Wilhelm Leibniz Universität Hannover, 30167 Hannover, Germany; pepelanova@iftc.uni-hannover.de (I.P.); katharina.kruppa@gmx.de (K.K.); scheper@iftc.uni-hannover.de (T.S.)

**Keywords:** 3D cell culture, GelMA, hydrogels, bioprinting, adipose tissue-derived mesenchymal stem cells (AD-MSCs), ASCs

## Abstract

Gelatin-methacryloyl (GelMA) is a semi-synthetic hydrogel which consists of gelatin derivatized with methacrylamide and methacrylate groups. These hydrogels provide cells with an optimal biological environment (e.g., RGD motifs for adhesion) and can be quickly photo-crosslinked, which provides shape fidelity and stability at physiological temperature. In the present work, we demonstrated how GelMA hydrogels can be synthesized with a specific degree of functionalization (DoF) and adjusted to the intended application as a three-dimensional (3D) cell culture platform. The focus of this work lays on producing hydrogel scaffolds which provide a cell promoting microenvironment for human adipose tissue-derived mesenchymal stem cells (hAD-MSCs) and are conductive to their adhesion, spreading, and proliferation. The control of mechanical GelMA properties by variation of concentration, DoF, and ultraviolet (UV) polymerization conditions is described. Moreover, hAD-MSC cell viability and morphology in GelMA of different stiffness was evaluated and compared. Polymerized hydrogels with and without cells could be digested in order to release encapsulated cells without loss of viability. We also demonstrated how hydrogel viscosity can be increased by the use of biocompatible additives, in order to enable the extrusion bioprinting of these materials. Taken together, we demonstrated how GelMA hydrogels can be used as a versatile tool for 3D cell cultivation.

## 1. Introduction

Recapitulation of the in vivo-like microenvironment during in vitro cell cultivation remains a challenge for researchers. Several important parameters/factors are usually neglected in conventional cell culture. These include physiological concentrations of biologicals (e.g., cytokines), energy sources (e.g., glucose and fatty acids), as well as oxygen concentrations. Moreover, cultivation in a three-dimensional (3D) microenvironment which supports cell-cell and cell-matrix interactions is still missing in most cell cultivation experiments. Several in vitro 3D cell culture systems have been developed in order to address this issue: cell aggregates, cell cultivation on natural and synthetic matrices and the encapsulation of cells in hydrogels [1].

Ever since its first description by Van Den Bulcke et al., gelatin methacryloyl (GelMA) has proved itself as one of the most versatile hydrogels available for 3D cell culture and bioprinting [2,3]. GelMA is a typical semi-synthetic hydrogel, which enables the exploitation of the biological signals inherent in the gelatin molecule, while allowing control of mechanical properties [1]. The hydrogel is obtained by the derivatization of gelatin with methacrylic anhydride, resulting in modification of lysine and hydroxyl residues with methacrylamide and methacrylate side groups [4]. After derivatization, the parent molecule gelatin retains many of its attractive features as a biomaterial: it still displays thermoreversible physical crosslinking and retains its biological promoting properties, based on integrin-binding sequences and metalloprotease digestion sites. The GelMA hydrogel can thus provide an aqueous environment for cells and supports their adhesion, growth, and proliferation. In contrast to gelatin, however, the modification with methacryloyl side groups allows the GelMA molecule to undergo rapid polymerization in the presence of UV light and a photoinitiator (PI), resulting in covalent crosslinking through the creation of a methacryloyl backbone [4]. This feature gives GelMA stability at physiological temperature and allows fine-tuning of mechanical properties. Moreover, the resulting material is transparent, which facilitates microscopic analysis.

GelMA is thus distinguished by its excellent biocompatibility, degradability, and low cost. Due to these features, GelMA has been extensively described for 3D cell culture applications and as a tissue engineering platform [5]. Although many studies using GelMA have been published in the last years, intensive literature research reveals great heterogeneity in material properties, concentrations and polymerization processes used to create these hydrogels. In our opinion, what is frequently missing is a systematic approach how to adapt GelMA to a given cell type and intended application. Frequently, existing studies do not answer the question of how exactly different parameters of polymerization influence final hydrogel properties and, consequently, the cell growth which occurs inside such constructs. In this work, we present a workflow that tailors the mechanical properties of the GelMA molecule to create the desired mechanical and architectural in vitro microenvironment. We have used adipose tissue-derived mesenchymal stem cells (AD-MSCs) as a model, since these cells are mechanosensitive, possess high motility, and are frequently used in tissue engineering applications.

Micro-molding with the hydrogel of choice is usually sufficient for most straightforward 3D cell culture applications. However, in order to introduce a higher degree of complexity, spatial control and resolution within an engineered construct, biofabrication techniques like lithography and rapid prototyping are becoming increasingly commonplace. As a bioink/biomaterial, GelMA shares a drawback with many other common hydrogels, in that it displays low viscosity resulting in poor resolution of printed structures, or even complete lack of printability at low concentrations with the most commonly used extrusion-based bioprinters [6].

To circumvent this problem, most published references print with higher concentrations of GelMA possessing high degree of functionalization (DoF) (higher crosslinking density) [7,8]. However, cells proliferate and migrate better when not hindered by a dense polymer network. Cell spreading and long term survival, as well as remodeling of the construct, cannot be observed in such constructs [9]. There are some strategies to approach the problem of poor printability of GelMA. The first method, followed for example by Billiet et al., uses the inherent thermo-reversible properties of GelMA and prints at temperatures lower than 37 °C. Shape fidelity is further supported by cooling the printing platform, which supports the natural sol-gel transition of GelMA [8].This approach is feasible, but applicable to high GelMA concentrations only (leading to hindered cell adhesion and migration as mentioned above) and limited by the fact that many low-cost extrusion bioprinters do not have temperature-controlled printing platforms.

Two additional methods for improving GelMA printability were demonstrated by the group of Schuurman et al. and include thermoplastic co-deposition or the mixing of hyaluronic acid into the bioink [10]. Many groups follow the latter approach and employ various rheological modifiers for enhancing GelMA deposition. Rheological modifiers are also sometimes called rheological additives and include components which enhance the rheology of the resulting bioink, especially with regard to viscosity and yield stress. Currently, there is no consensus for which rheological modifier works best for GelMA bioprinting. Good results have been obtained with materials as diverse as gellan gum [11] and nanoparticles [12]. The modifier may be selected based solely on its viscosity-enhancing properties (e.g., alginate) or depending on the final application (e.g., calcium phosphate for bone tissue engineering). In any case, fine-tuning is mandatory in order to close down on the optimal biofabrication window [9]. While viscosity can be increased by the use of rheological modifiers, care should be taken that the resulting hydrogel mixture can still support cell adhesion, spreading, and proliferation.

In this work, we share our experiences on how GelMA can be established as a general all-round platform for 3D cell culture and bioprinting. Using accessible bioprinting equipment and cost-efficient protocols, it is our conviction that any lab can establish an economic and versatile platform for 3D cell cultures of varying complexity based on GelMA. We will cover how GelMAs of specific DoF can be produced and characterized. These materials should then be screened together with the cell type of interest in order to determine which material is best suited to the intended cell type. Moreover, the subsequent analysis of the total cell population inside the hydrogel construct may require cell release. Therefore, we will also cover the enzymatic degradation of GelMA. Finally, we describe additives which are applicable for extrusion-based bioprinting techniques, and discuss their advantages and limitations.

## 2. Methods

### 2.1. GelMA Synthesis

Gelatin methacryloyl of various DoFs was synthesized by the method of Shirahama and Lee [13]. The A100 high DoF material was synthesized via the one-pot method [13], while the lower DoF materials were obtained by the sequential pH adjustment method [14]. Briefly, a 10% (w/v) gelatin solution of type A (porcine, Bloom strength 300, Merck KGaA, Darmstadt, Germany) or type B (bovine, Bloom strength 225, Merck KGaA, Darmstadt, Germany) was dissolved under stirring in a pH 9, 0.1 M carbonate-bicarbonate buffer (CB buffer, Merck KGaA, Darmstadt, Germany) at 60 °C. The reaction was started by the addition of methacrylic anhydride (MAA, Merck KGaA, Darmstadt, Germany) at 50 °C and with rigorous stirring at 500 rpm. MAA was added with sequential steps, giving a total MAA amount equal to the defined ratio in each recipe (x mL MAA/g gel, see Table 1, Results). The pH was adjusted back to 9 after each addition. The reaction was carried out for 60 min and terminated by pH adjustment to 7.4. The A100 material was synthesized in a 0.25 M CB buffer with a 0.1 MAA/g Gel ratio, by performing a single MAA addition and pH adjustment. After completion, the reaction mixture was filtered using standard paper filters (Whatman™, 90 mm diameter, GE Healthcare Life Sciences, Little Chalfont, Buckinghamshire, UK) and dialyzed with a 14 kDa molecular-weight-cutoff (MWCO) membrane (Carl Roth GmbH & Co. KG, Karlsruhe, Germany) at 40 °C for 36 h against ultrapure water. Water was changed every 4 h to ensure complete removal of low molecular weight gelatins and methacrylic reaction byproducts. GelMA solution after dialysis was frozen and lyophilized. The resulting GelMA product was stored in the dark at 4 °C.

### 2.2. DoF Determination

The degree of functionalization (DoF) was determined by the trinitrobenzenesulfonic (TNBS) acid method based on Habeeb [15] and modified by Lee and Shirahama [13]. Briefly, samples and underivatized gelatin A & B were dissolved in 0.1 M CB buffer (90 mg/100 mL). A 500 µL sample was mixed with 500 µL reagent (0.01% TNBS in 0.1 M CB buffer) and incubated for 2 h at 37 °C. The reaction was stopped by the addition of 250 µL HCl (1 M) and 500 µL SDS (10%). The resulting UV absorption was measured at 335 nm with a Multiskan GO spectrophotometer (Thermo Fischer Scientific, Waltham, MA, USA) and quantified by using a glycine calibration curve.

### 2.3. Hydrogel Preparation

GelMA solutions were prepared at the indicated concentrations by weighing an appropriate amount of GelMA and dissolving it in phosphate-buffered-saline (PBS, pH 7.4). The GelMA solution was incubated in a water bath until complete dissolution of the GelMA solid and disappearance of foam at 37 °C. All GelMA solutions contained 2-hydroxy-4′-(2-hydroxyethoxy)-2-methylpropiophenone (Irgacure 2959) at a final concentration of 0.1% (w/v). Prior to cell experiments, warm GelMA solutions were sterile-filtered using 0.45 µm polyethersulfone (PES) filters (10 mm diameter) in a laminar-flow cabinet.

### 2.4. Rheological Characterization

The viscosity of GelMA solutions was determined at 25 °C by rotational viscosimetry using a MCR 302 Modular Rheometer (Anton Paar, Graz, Austria) equipped with plate-plate geometry (40 mm plate diameter) and a gap size of 0.4 mm. The shear rate was varied from 0.01 to 1000 s^−1^, which is a typical shear rate region associated with pneumonic extrusion bioprinting.

The UV polymerization of GelMA solutions was investigated at 25 °C using a MCR 302 Modular Rheometer (Anton Paar, Graz, Austria) equipped with a plate-plate geometry (20 mm diameter, 0.3 mm gap size) and by performing in situ UV crosslinking by irradiation with a UV lamp (Delolux 80, Delo, Windach, Germany) from below. The crosslinking was recorded with a time sweep oscillatory test under constant strain amplitude of 1% and at a constant frequency of 1 Hz, which is within the linear viscoelastic (LVE) region. The sample volume was 100 µL, and the UV dosage was varied as indicated in the results.

### 2.5. Cell Culture

Human adipose tissue-derived mesenchymal stem cells (hAD-MSCs were isolated from the adipose tissue of four donors after abdominoplasty. All patients have given their informed consent, as approved by the Institutional Review Board (Hannover Medical School). The isolated cell populations have been extensively characterized as mesenchymal stem cells by surface marker analysis and functional properties [16]. Briefly, cells were characterized by flow cytometry for specific immunotypic MSCs markers and were positive for CD44, CD73, CD90, and CD105 and negative for hematopoetic (CD34 and CD45) and endothelial (CD31) markers. Furthermore, hAD-MSCs were able to differentiate under controlled conditions toward adipocytes, chondrocytes, and osteocytes (confirmed by BODIPY, Alcian Blue and Alizarin red stainings respectively). AD-MSCs were expanded in alpha-MEM medium (Thermo Fisher Scientific, Waltham, MA, USA) containing 1 g/L glucose, 2 mM l-glutamine, 10% human serum (CC-pro, Oberdorla, Germany) and 50 µg/mL gentamicin (Merck KGaA, Darmstadt, Germany), harvested by accutase treatment (Merck KGaA, Darmstadt, Germany) and cryopreserved at passage two until the start of the experiment. Experiments were performed with cells of passages two to eight.

### 2.6. 3D Cell Culture in GelMA Hydrogels

For 3D cell culture experiments, 1.5 × 10^6^ cells/mL hydrogel were encapsulated in 50 µL disks (6 mm diameter) in silicon molds with the help of a cross linker (BLX-365 BIO-LINK, 365 nm, Vilber Lourmat Deutschland GmbH). UV intensity varied between 1.2 J/cm^2^ and 2.4 J/cm^2^. Polymerized hydrogel constructs were incubated in 24-well plates in 400 µL alpha-MEM medium (Thermo Fisher Scientific, Waltham, MA, USA) containing 1 g/L glucose, 2 mM l-glutamine, 10% human serum (CC-pro, Oberdorla, Germany), and 50 µg/mL gentamicin (Merck, Darmstadt, Germany).

### 2.7. Enzymatic Digestion of GelMA with and without Cells

To measure the enzymatic digestion of GelMA with various DoFs, 50 µL disks (6 mm in diameter) were made using a silicon mold in a Petri dish under sterile conditions. GelMA hydrogel solution was dispensed into the mold and subsequently polymerized with a cross linker (BLX-365 BIO-LINK, 365 nm, Vilber Lourmat Deutschland GmbH, Eberhardzell, Germany). After polymerization, the hydrogel discs were transferred to 24-well-plates and allowed to swell overnight in 500 µL of alpha-MEM medium supplemented with 10% human serum (CC-pro, Oberdorla, Germany) and 50 µg/mL gentamicin (Merck, Darmstadt, Germany). After swelling of the constructs, cell culture medium was removed and 500 µL of collagenase-CLS I with 20 or 30 U/mL (Merck, Darmstadt, Germany) in Hanks Balances Salt Solution (Sigma Aldrich (now Merck KGaA, Darmstadt, Germany) supplemented with 3 mM CaCl_2_ were added to each well. Hydrogels in collagenase-CLS I solution were placed in an incubator equipped with a rotary shaker and incubated for over 5 h (130 rpm, 37 °C). At the given time point, constructs were removed from the enzymatic solution and excessive liquid was carefully removed by blotting dry with a delicate tissue wipe (Kimtech Science, Kimberly-Clark, Irving, TX, USA). The degree of degradation was estimated by the measurement of hydrogel weight loss in comparison to the original wet weight after swelling. Four separate constructs per time point and digestion condition were used in this experiment.

### 2.8. Cell Viability and Proliferation

The viability and proliferation of AD-MSCs in the GelMA hydrogels was measured indirectly by the Cell Titer-Blue® (CTB) cell viability assay (Promega, Mannheim, Germany). Briefly, cells were encapsulated in the hydrogels by polymerization in a UV-cross linker (BLX-365 BIO-LINK, 365 nm, Vilber Lourmat Deutschland GmbH, Eberhardzell, Germany) at different UV intensities. After polymerization, the cell-laden constructs were placed in 24-well-plates and 400 µL alpha-MEM medium supplemented with 10% human serum (CC-pro, Oberdorla, Germany) and 50 µg/mL gentamicin (Merck, Darmstadt, Germany) was added to each well. On day 1 (24 h), day 3 (72 h), and day 7 (168 h) of cultivation, cell culture medium was removed and 400 µL CTB working solution (1:10 (*v*/*v*) in basal alpha-MEM) were added to each well. After 5 h of incubation, CTB fluorescence was measured at an extinction wavelength of 544 nm and an emission wavelength of 590 nm using a microplate reader (Fluoroskan Ascent, Thermo Fisher Scientific Inc., Waltham, MA, USA). Values are presented as a direct CTB fluorescence signal after blank subtraction (400 µL CTB working solution (1:10 (*v*/*v*) in basal alpha-MEM) after 5 h incubation in the absence of cells and hydrogel). At least four constructs per material were measured for a single time point.

### 2.9. Live/Dead Staining

For representative live/dead staining, as well as for morphological examination, cell-laden hydrogels (one construct per time point and per material) were incubated in 3 µm Calcein-AM (Merck, Darmstadt, Germany) and 2.5 µm propidium iodide (Merck, Darmstadt, Germany) solution in basal alpha-MEM for 15 min at 37 °C and analyzed with a fluorescent microscope (Olympus, IX50, Olympus Corporation, Tokyo, Japan), equipped with a camera (Olympus SC30, IX-TVAD, Olympus Corporation, Tokyo, Japan) and the CellSens Software (CellSens Standard 1.7.1, Olympus Corporation, Tokyo, Japan).

### 2.10. Bioprinting

GelMA hydrogel was prepared and mixed with additives at the desired concentration. The hydrogel was filled in a sterile disposable syringe and inserted into an extrusion bioprinter (BioBot 1, Allevi, Philadelphia, PA, USA). The printing needle diameter (Nordson EFD, Oberhaching, Germany) was 0.40 mm, the extrusion air pressure ranged between 2.8 and 3.8 psi, and printing was performed at 30 °C or 37 °C with a deposition speed of 260 mm/min. A diagonal grid pattern of 2.5 cm × 2 cm dimensions was printed with and without curing by a UV cross linker (BLX-365, BIO-LINK 365 nm, Vilber Lourmat Deutschland GmbH, Eberhardzell, Germany). Micrographs of the printed constructs were taken with a Stereo zoom microscope BMS 133, (BMS microscopes b.v., Capelle aan den Ijssel, the Netherlands). Alginate-2-Hydroxyethyl Methacrylate (AlgHEMA) was provided by Petersburg State University, Saint Petersburg, Russia. Silicate nanoparticles (SiNP), trade name Laponite XLG, were provided by BYK Additives Ltd., Widnes, UK.

## 3. Results

### 3.1. GelMA Synthesis and Characterisation

**Figure 1:**
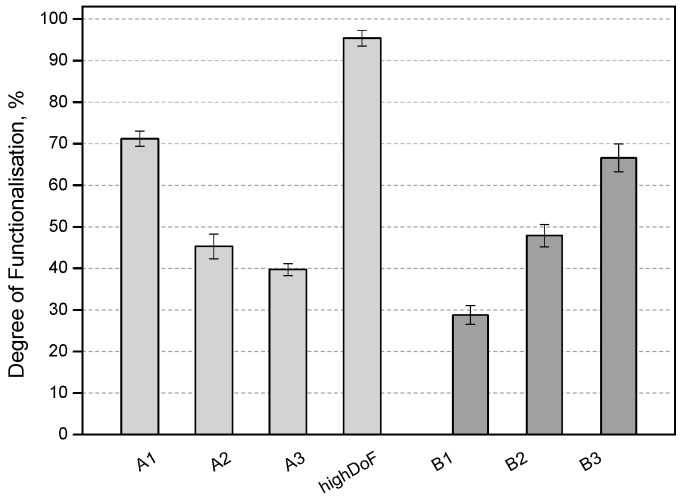
GelMA materials of various DoFs produced by the Shirahama-Lee method and the implementation of a low MAA to gelatin ratio with both type A and B gelatin. The DoF was determined by the TNBS assay.

Different batches of GelMA were synthesized from both types A and B gelatin by a variation of the ratio between methacrylic anhydride (MAA) and gelatin (Figure 1). The method of derivatization used in our study, displayed various advantages in comparison to the traditional protocol in PBS (pH 7.4). Whereas traditional protocols employ 10–32 molar excess of MAA, which is a volatile, toxic and irritant organic compound, the current method used only a low ratio between MAA and gelatin to obtain specific DoFs. The Shirahama-Lee method of derivatization uses a pH of 9 for the reaction, which is above the isoelectric point of both type A and B gelatins. This ensures that the lysine side groups of the protein remain neutral and are available for the reaction with MAA. The pH of the reaction sinks constantly due to the production of methacrylic acid, requiring manual or automatic pH adjustment of the reaction back to 9, thus ensuring optimal reaction conditions which necessitate lower amounts of MAA and result in specific DoFs of the product. Table 1 gives an overview of the different GelMA materials produced, which include highly derivatized materials (A70, B70, A100), low derivatized materials (B30, A40) and materials in the middle range of derivatization (A50, B50).

Both type A and B gelatins may be used for functionalization of GelMA and yield a cell-compatible material. The Bloom factor of the parent gelatin molecule is an important factor to consider, since it describes the gel strength of the gelatin and will influence the gel strength of the GelMA obtained. Type B material is derived from bovine skin and alkaline treatment. As a result, it has a more fragmented structure and chains of lower molecular weight (lower Bloom strength factor). This means that after derivatization, B materials will be softer than A materials of the same DoF. Type A gelatin is derived from porcine skin after acid treatment; it has a higher transparency than type B materials and can be obtained with a maximum Bloom factor of 300, also used in this work.

**Figure 2:**
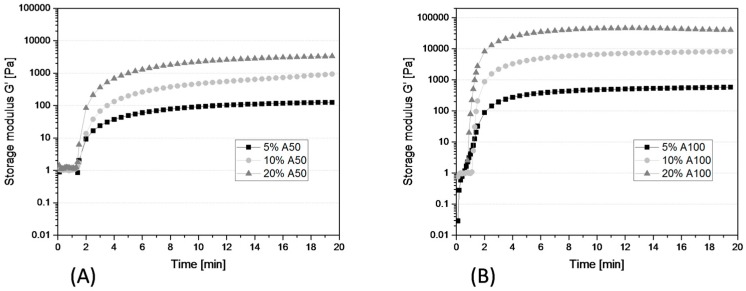
Crosslinking kinetics of A50 (**A**) and A100 (**B**) in various concentrations measured in a time sweep experiment. Shown is G’ (storage modulus) evolution with time; G’’ (loss modulus) is not shown for the purpose of clarity; in all samples G’ > G’’ after illumination, indicating a well-developed and solid polymer network. Samples were measured at 25 °C and were illuminated with an ultraviolet (UV) intensity of 1.2 J/cm^2^, (representative curves of three measurements).

Having a range of GelMA materials allows systematic screening for which material is best suited as a 3D microenvironment to the specific cell type studied. The resulting mechanical properties of GelMA will depend on the starting gel strength of the parent gelatin molecule (Bloom factor), the DoF obtained after derivatization (resulting in higher or lower crosslink density), the hydrogel concentration used in the experiment, and the UV polymerization conditions. As an illustration of this effect, Figure 2 shows the UV polymerization of various concentrations of the A50 and A100 material, as viewed in time sweep rheological experiments. As can be expected, an increase in the GelMA concentration led to a higher storage modulus (G’) and stiffer constructs in both material types, A50 and A100. The higher derivatization in the A100 material corresponded to a higher crosslinking density, which resulted in stiffer hydrogels for A100 in comparison to A50 at the same concentration. Also, the higher density of methacryloyl groups in A100 led to a slightly faster start of polymerization, all other UV polymerization conditions such as light intensity and PI concentration being equal in both cases.

### 3.2. Cell Culture Experiments

Fine-tuning of the mechanical properties of GelMA depends on the cell culture application intended. In this work, a material was required that would support the adhesion, spreading, and proliferation of MSCs, and that would display sufficient mechanical stability for extrusion bioprinting at the same time. The rheological characterization of the different GelMA batches, as well as the physical handling of the hydrogels revealed that materials in the middle and high range of DoF (DoF of 50 and above) would probably fit this fabrication window, while the low DoF materials (B30, A40) were too soft for reliable handling and bioprinting. Therefore, cell culture experiments were performed with the A50, A70, B50, B70, and A100 batches. For this purpose, cells were encapsulated in 5% (w/v) hydrogels by illumination with UV light, and the viability of the cells was monitored using CTB assay and live/dead staining. This step was performed to evaluate a possible toxic effect of derivatization side-products (e.g., via insufficient dialysis) and potential damage during UV-curing.

**Figure 3:**
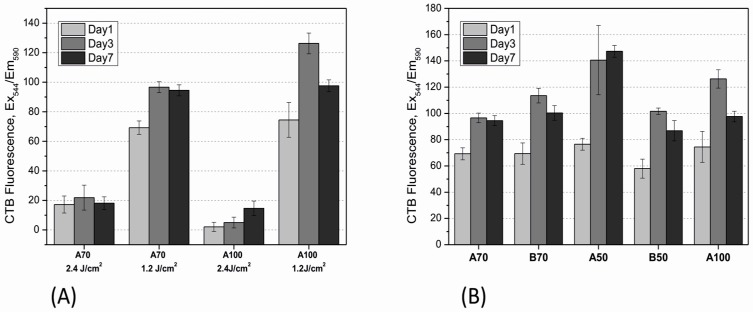
(**A**) the influence of UV intensity (1.2 J/cm^2^ and 2.4 J/cm^2^) during polymerization on the viability of human adipose-derived mesenchymal stem cells (hAD-MSCs) encapsulated in GelMA hydrogels. (**B**) Cell viability in various GelMA hydrogels after polymerization at 1.2 J/cm^2^; CTB assay was performed on day 1, day 3, and day 7 of cultivation. GelMA conc. 5% (w/v).

To evaluate the influence of UV intensity on the viability of encapsulated cells, hydrogels with different degrees of functionalization (A70 and A100) were crosslinked with either 2.4 J/cm^2^ or 1.2 J/cm^2^. Cell viability was estimated indirectly with the aid of a CTB assay on day 1, day 3 and day 7 (Figure 3). As can be seen, a higher UV intensity led to lower cell viability after encapsulation. Both studied materials demonstrated increased cell viability after polymerization with 1.2 J/cm^2^ in comparison to 1.4 J/cm^2^ (Figure 3A). Apart from the higher UV dose, it was likely that the 2.4 J/cm^2^ illumination conditions also led to different material properties, which are less favorable for MSCs. To evaluate this hypothesis, the stiffness of the hydrogels resulting from each UV dose was determined by rheology.

**Figure 4:**
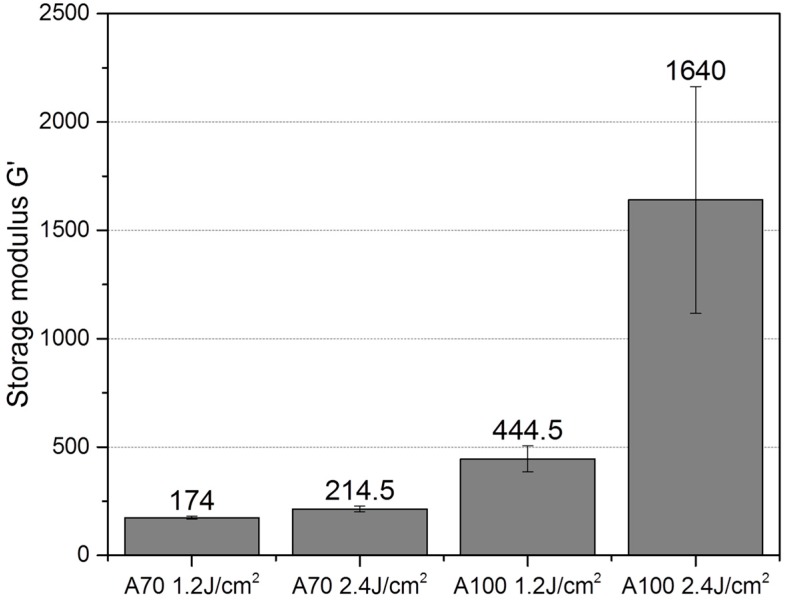
Material stiffness of GelMA hydrogels (5% w/v) at 25 °C crosslinked with 1.2 J/cm^2^ or 2.4 J/cm^2^, determined by in-situ polymerization in a time-sweep oscillatory test.

Figure 4 shows that, all other conditions like hydrogel and PI concentration being equal, the higher UV dosage did lead to a stiffer material. This effect was especially pronounced for the A100 material and less pronounced but significant for the A70 material. Beyond stiffness, additional effects such as pore size and architecture, or lower amount of generated radicals during photopolymerization might play a role why MSCs exhibit higher viability in the material produced with lower UV dosage.

**Figure 5:**
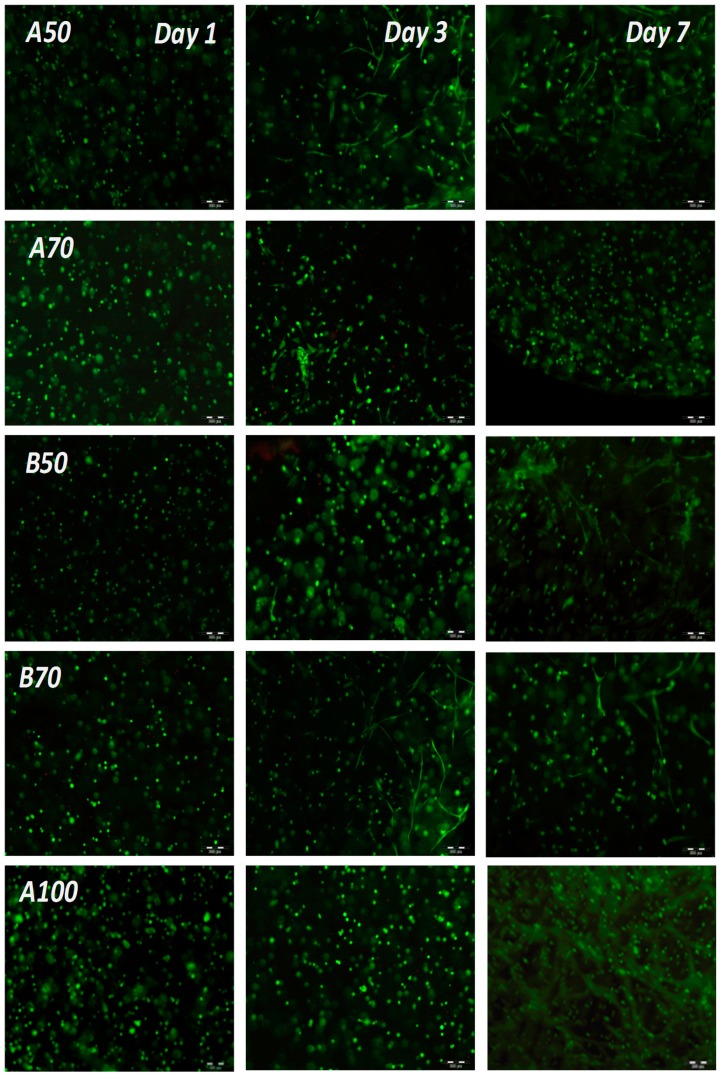
Live/dead staining and morphological examination of hAD-MSCs encapsulated with a UV dose of 1.2 J/cm^2^ in 5% GelMA with various DoF. Calcein-AM/Propidium iodide, 4× objective, scale bar 200 µm.

The application of lower UV intensity (1.2 J/cm^2^) for polymerization of the other materials (B70, B50 and A50) also supported high cell viability over the entire period of cultivation (Figure 3B). The highest signal was obtained in A50, demonstrating that this DoF probably displays the optimal pore size and stiffness for cell growth. These findings were also confirmed by monitoring of cell morphology. In addition to the CTB assay, calcein-acetoxymethyl (AM)/propidium iodide staining was performed. As shown in Figure 5, the live/dead staining indicated that cells in all tested materials displayed high viability over the entire period of cultivation, with only very few dead cells (red stained) observed. Moreover, the cells could spread and establish cell-cell contacts in both (A and B) GelMA hydrogels with 50% functionalization. Cells encapsulated in GelMA B70 with a 70% DoF also demonstrated spreading; however, it was less pronounced than the cell spreading observed in B50 and A50. Cells encapsulated in GelMA A hydrogels with high derivatization A100 and A70 remained round, without cell-cell contacts. Cell viability, however, remained high. It is also interesting to note that higher hydrogel concentrations of all materials (e.g., 10% w/v) did not support hAD-MSC spreading anymore, showing that the hydrogel concentration is a critical factor in this regard (data not shown).

### 3.3. GelMA Digestion

**Figure 6:**
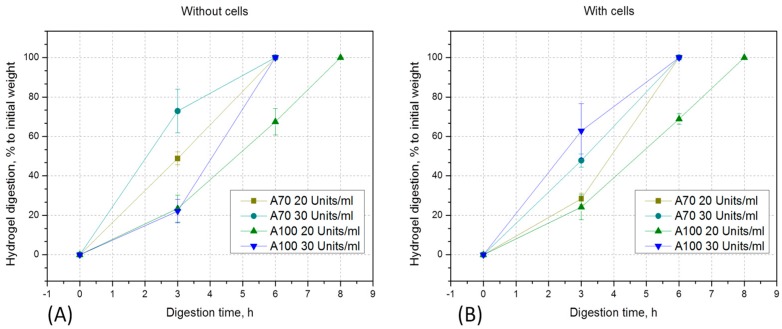
Enzymatic degradation of A100 and A70 GelMA, 10% (w/v) with 20 U/mL or 30 U/mL collagenase-CLS I: (**A**) gel constructs without cells, (**B**) gel constructs with encapsulated cells.

One of the challenging issues of cell cultivation in hydrogels is the possibility of cell release from the polymerized constructs for closer analysis (e.g., PCR, intracellular proteins quantification or flow cytometry). In order to study the option of controllable hydrogel degradation in vitro, collagenase digestion of polymerized constructs with and without cells was performed (Figure 6). Already after 3 h of incubation with collagenase, 45% (20 U/mL) and 75% (30 U/mL) digestion of A70 gels in the absence (Figure 6A), and 45% (20 U/mL) and 65% (30 U/mL) in the presence of cells (Figure 6B), was observed. In A100 hydrogels, only about 20% digestion (in 20 U/mL and 30 U/mL both) was measured with and without encapsulated cells after 3 h. A70 GelMA hydrogels with and without cells were fully digested after 6 h of incubation in 20 U/mL and 30 U/mL of collagenase. High DoF GelMA could also be completely digested in 30 U/mL collagenase after 5 h, but in 20 U/mL collagenase only after 8 h of incubation (again both, with and without cells). As can be seen, the speed of digestion was inversely correlated with the DoF. Lower derivatized materials were degraded even faster than the high DoF materials described in this experiment. The kinetics of digestion will also naturally depend on the hydrogel concentration and collagenase I amount. It is also important to note that after digestion, cells retained viability and could adhere on the plastic surface of the well (data not shown).

### 3.4. Extrusion Bioprinting

**Figure 7:**
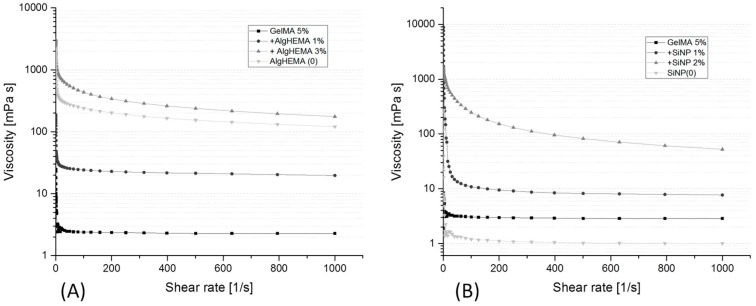
Rheological characterization of GelMA by rotational viscosimetry, (**A**) GelMA (5% w/v) at 37 °C with various concentrations of alginate derivatives (AlgHEMA) (w/v) as an additive and a negative control of AlgHEMA 3% w/v (0), (**B**) GelMA (5% w/v) at 30 °C with various concentrations of silicate nanoparticles (SiNPs) as additives, and a negative control of SiNPs 2% w/v only (0), (*n* = 3).

From all materials screened in this study, A50, B50 and B70 were shown to support hAD-MSC proliferation and spreading best at the concentration of 5% (w/v) and by crosslinking with 1.2 J/cm^2^. All three materials can be adapted as bioinks for extrusion bioprinting. However, on their own, each of these hydrogels has very low viscosity at the used concentration (see Figure 7), leading to poor printability. Decreasing the printing temperature below 30 °C increased viscosity, but impeded and complicated printing due to frequent clogging of the printing needle. At temperatures below 30 °C thermoreversible sol-gel transition of GelMA occurs, leading to partial gelation and inhomogeneity of the biomaterial. Clogging and pressure fluctuations make the bioprinting difficult to reproduce. In order to approach the biofabrication window, different additives were added to the GelMA and the resulting viscosity was examined by rotational viscosimetry. Themost promising results with the extrusion bioprinter were obtained by alginate derivatives (AlgHEMA) and by silicate nanoparticles (SiNPs).

In both cases, an increase of the additive amount led to an increase in the viscosity observed at all shear rates. The presence of additives led to pronounced shear thinning behavior, with high viscosity at low shear rates and lower viscosities at high shear rates. Shear-thinning (pseudoplastic) behavior is a requirement for hydrogel bioinks as the higher shear rates present in the printing needle during extrusion lead to easier filament deposition, while the low rates after printing support high shape fidelity. Two different mechanisms of viscosity increase were present in this case. The AlgHEMA polymer simply acted as a water binding agent of high molecular weight. The resulting viscosity of the GelMA/AlgHEMA was the sum of the viscosities of both components (Figure 7A). In contrast, the viscosity-enhancing mechanism of the SiNP particles was based on an electrostatic interaction of the nanoparticles with the GelMA chains. Therefore, the resulting viscosity of the GelMA/SiNP was higher than the sum of individual components (viscosity of GelMA or SiNP only, Figure 7B).

**Figure 8:**
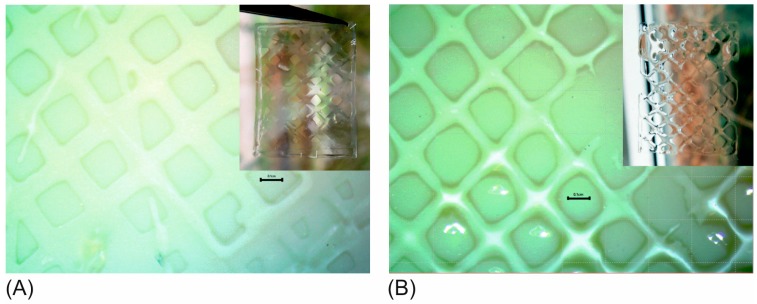
Lattices of dimensions 2 × 2.5 cm printed with (**A**) bioink composed of GelMA and 3% AlgHEMA printed at 37 °C with extrusion pressure of 3.8 psi, nozzle speed 260 mm/min, and (**B**) bioink of GelMA and 1% SiNPs printed at 30 °C with extrusion pressure of 2.8 psi, nozzle speed 260 mm/min. Structures shown after UV crosslinking.

Figure 8 shows constructs printed with the GelMA containing either AlgHEMA (Figure 8A) or SiNP (Figure 8B) as additives. In both cases, structures with high fidelity and good printability could be obtained. Whenever possible, direct printing was performed at 37 °C. It was possible to decrease the additive concentration by using a slight decrease in temperature and still obtain good printability (Figure 8B). Here, a bioink with 1% SiNP was printed at 30 °C.

## 4. Discussion and Conclusions

The attempt to approach physiological conditions in in vitro experiments plays an important role for the better understanding of cell physiology, cell-matrix interactions, and intercellular communication. Moreover, 3D cell models allow better evaluation of drug candidates, which helps with prediction of treatment outcomes before starting animal trials, thus saving costs and reducing the number of animal experiments required. Numerous original studies and reviews have shown great differences in cell reactions between two-dimensional (2D) and 3D cell cultures, and the importance of creating a more physiological in vitro cell microenvironment [17,18,19]. Additive manufacturing technologies (bioprinting) represent an advanced technique of 3D cell culture. Bioprinting brings 3D cell culture to the next level by allowing spatial control of construct architecture. Thus, it is possible to print different materials (e.g., with variable mechanical stiffness or pore size) with different cells for heightening the complexity of the cell models of interest

The possibility of precisely tuning and adapting hydrogels to the intended application provides researchers with a valuable tool for the creation of specific in vitro microenvironments. From this point of view, GelMA provides a perfect cultivation platform: (1) it can be easily synthesized in the lab for a low price, (2) it is transparent (convenient cell monitoring), (3) it has RGD motifs for cell adhesion, (4) its concentration can be varied in order to achieve a desired stiffness, (5) its DoF can be also adapted to create hydrogels with particular stiffness and pore size and (6) it can be digested in a controllable manner if cell analysis is required after cultivation. 

**Figure 9:**
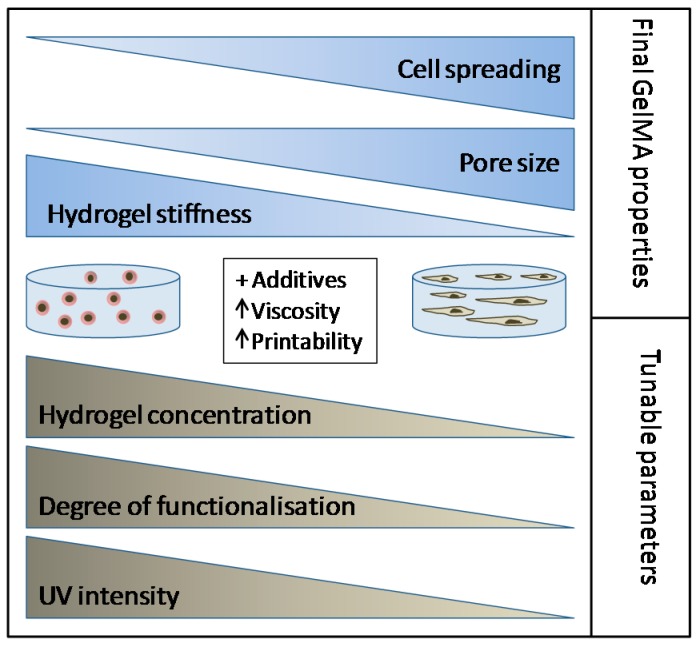
GelMA as a toolbox for tunable hydrogel constructs and bioprinting.

Using the GelMA toolbox, researchers can either identify optimal hydrogel conditions for encapsulation of the cells of interest, or may manipulate the scaffold for the study of the influence of microenvironment on cell fate. Since different tissues have different mechanical properties, combination of specific concentrations, DoFs and UV polymerization dosages can be used to approach the stiffness of every soft tissue (Figure 9).

In this work, we aimed to adapt GelMA as a 3D cell culture platform and as a bioink for the cultivation of hAD-MSCs. The encapsulation of cells in restrictive GelMA microenvironments of high stiffness for the purpose of bioprinting [7,8,10,20], microtissues [21], or tissue engineering [10] is well-described in the literature. In contrast, reports of the adaptation of GelMA to constructs that do not restrict cell spreading and migration are more rare (for example, the 3D GelMA cancer models of Kaemmerer et al. [22]). We showed that a low polymer concentration of GelMA and a low UV dosage are essential for creating a cell promoting microenvironment for MSCs. Encapsulated cells displayed excellent viability under these conditions.

There are two general ways in which cell physiology can be affected in the resulting 3D GelMA construct. First, the UV polymerization conditions themselves (such as UV light intensity and PI) may affect cell viability. Second, the architecture of the construct, especially pore size, may affect oxygen and nutrient diffusion, as well as inhibiting migration and intercellular interactions. In this work, cells were studied 24 h after encapsulation and after three days and seven days of cultivation. Higher UV intensity (2.4 J/cm^2^) resulted in lower cell viability in comparison to cells encapsulated in the same material at 1.2 J/cm^2^. The lower cell viability after higher UV dosage exposure might not necessary be the result of direct cytotoxicity. Earlier, Fedorovich et al. demonstrated that UV polymerization can be toxic to MSCs in 2D cultures, but has no effect on cells encapsulated in hydrogels [23]. This can be explained by the fact that the radicals produced in the reaction are usually captured by unreacted methacrylate groups, which exert a protective effect on encapsulated cells. On the other hand, UV irradiation doses of 2.7 J/cm^2^ have been linked with decreased viability in encapsulated cells in comparison to lower UV intensities. Billiet et al. showed that this decreased viability is probably related to both- the denser network properties and the higher amount of generated free radicals under this illumination conditions [8].

We could also show that a higher UV dosage leads to a dramatic increase of stiffness in high-DoF materials. The increase was not as significant in constructs of 70% DoF. Here, additional effects such as final pore size may play a role in the explanation as to why cell spreading does not occur at this crosslinking intensity. For example, Chen et al. demonstrated that the pore size in 49.8% DoF materials is significantly larger than in 73.2% DoF materials. This group could also show the generation of 3D vascular networks with GelMA and also employed low polymer concentration and UV intensity to achieve cell spreading (5% w/v GelMA and 0.134 J/cm^2^) [24]. Further studies, however, must be performed in order to understand how material stiffness and pore size work together to create promoting cell microenvironments. To our knowledge, there are no reliable methods available to estimate the pore size of wet hydrogels.

Cells encapsulated in A50 and B50 showed good spreading. However, higher viability was observed in A50 after seven days of cultivation. No spreading was detected in materials of DoF 70% and above. The reason could be a smaller pore size in high DoF GelMA constructs, which disturbs migration and cell-cell interaction. However, cell viability increased in all GelMA constructs during the course of a week. One reason for this observation could be cell growth on the surface of the constructs with high DoF. It is interesting to note, that hAD-MSCs proliferated much faster on the surface of materials with 70% DoF and higher. Indeed, it was demonstrated earlier for 2D cell cultures growing on the surface of hydrogels with different stiffness, that a soft matrix had a negative effect on cell proliferation in comparison to a stiffer matrix [25].

Although this work provides a detailed systematic approach for establishing a GelMA-based 3D cell culture, a deeper biological investigation into the cell biology after encapsulation must still be performed. The influence of hydrogel stiffness and architecture on the focal adhesion, Yes-associated protein expression, and cell migration in the hydrogels can be explored further. Another important issue would be a study of MSCs differentiation in hydrogels of different architecture, since stiffness has a great influence on mechanosensitive MSCs [26,27]. Our work, however, provides a fundament for further studies involving the creation of in-vitro platforms with variable stiffness, such as hydrogels with stiffness gradients.

Excellent studies describing various additives for GelMA bioprinting have been published in recent years, such as the use of gellan gum [11], nanosilicates [12], hyaluronic acid [10], and alginate [28]. There is no consensus for which additive works best, and considerations are focused on the intended application (like adding methacrylated hyaluronic acid (HAMA) as an extracellular matrix (ECM) component for cartilage tissue engineering [29,30]) or are limited by the availability of hardware specifics of the bioprinter equipment (like a coaxial extrusion system based on a microfluidic system [28]). Both unmodified and methacrylated biopolymers can be used for viscosity increase of the GelMA bioink. Here as well, further studies are needed in the field on order to evaluate whether covalent crosslinking between the polymers is necessary for long-term stability, or whether a simple inter-penetrating polymer network of GelMA and the polymer additive is sufficient for the fabrication purpose. To adapt GelMA as a bioink without compromising the cell promoting environment necessitates a careful formulation of additives to increase the viscosity, without impacting cell spreading and proliferation to a great extent. Our experience with different additives shows that SiNPs and the novel alginate-HEMA are good candidates for achieving this task.

## Figures and Tables

**Table 1:** Overview of the different batches of gelatin methacryloyl (GelMA) produced in this work and the nomenclature used to name them throughout this manuscript.

Product	Gelatin Type	Ratio (MAA (mL)/gel (g))	CB-Buffer	pH Control	DoF (%)	Name
GelMA A1	A	0.05	0.1 M	sequential	71.2	A70
GelMA A2	A	0.025	0.1 M	sequential	45.3	A50
GelMA A3	A	0.0375	0.1 M	sequential	39.7	A40
GelMA B1	B	0.025	0.1 M	sequential	28.8	B30
GelMA B2	B	0.0375	0.1 M	sequential	47.9	B50
GelMA B3	B	0.05	0.1 M	sequential	66.6	B70
GelMA high DoF	A	0.2	0.25 M	one-pot	95.4	A100
